# Garcinone E induces apoptosis and inhibits migration and invasion in ovarian cancer cells

**DOI:** 10.1038/s41598-017-11417-4

**Published:** 2017-09-06

**Authors:** Xiao-Huang Xu, Qian-Yu Liu, Ting Li, Jian-Lin Liu, Xin Chen, Li Huang, Wen-An Qiang, Xiuping Chen, Yitao Wang, Li-Gen Lin, Jin-Jian Lu

**Affiliations:** 1 State Key Laboratory of Quality Research in Chinese Medicine, Institute of Chinese Medical Sciences, University of Macau, Macao, China; 2 Guangdong Medical Device Quality Surveillance and Test Institute, Guangzhou, Guangdong, China; 3 Division of Reproductive Science in Medicine, Department of Obstetrics and Gynecology, Feinberg School of Medicine, Northwestern University, Chicago, Illinois USA; 4 Center for Developmental Therapeutics, Chemistry of Life Processes Institute, Northwestern University, Evanston, Illinois USA

## Abstract

Ovarian cancer remains the most lethal gynecological malignant tumor. In this study, 24 xanthones were isolated and identified from the pericarps of mangosteen (*Garcinia mangostana*), and their anti-proliferative activities were tested in ovarian cancer cells. Garcinone E (GE) was found to exhibit excellent anti-proliferative effects among the tested xanthones. It significantly inhibited the proliferation in HEY, A2780, and A2780/Taxol cells as evidenced by 3-(4,5-dimethylthiazol-2-yl)-2,5-diphenyl tetrazolium bromide (MTT) assay, lactate dehydrogenase (LDH) release assay, Hoechst 33342 staining, annexin V/PI staining, and JC-1 staining. It induced endoplasmic reticulum (ER) stress and activated the protective inositol-requiring kinase (IRE)-1α pathway. Knocking down IRE-1α further activated the caspase cascade and caused an increase in cell death. Moreover, GE eliminated the migratory ability of HEY cells by reducing the expression of RhoA and Rac. It also blocked the invasion, which might be related to downregulation of matrix metalloproteinases (MMPs), *i.e*., MMP-9 and MMP-2, and upregulation of tissue inhibitors of metalloproteinase (TIMP) -1 and TIMP-2. In summary, GE exerts anticancer activities by inducing apoptosis and suppressing migration and invasion in ovarian cancer cells, which indicates its therapeutic potential for ovarian cancer.

## Introduction

With a five-year survival rate that remained below 40% for decades, ovarian cancer is considered to be the most lethal gynecological malignant tumor in the world^1^. Among all ovarian tumor cases, 90% are diagnosed as epithelial ovarian cancer, and about 60% of them are further classified as serous carcinoma with highly heterogeneous characteristics. Surgery is the preferred option in the majority of ovarian cancer cases. However, high rates of metastasis in malignant stages make eradication of lesions by surgical therapy alone difficult, and chemotherapy is required for eliminating the lesions in most procedures^2^. While platinum-taxane doublet-based chemotherapy has remained as the first-line strategy in the past 20 years, chemo-resistance occurs in over 70% of ovarian cancer patients and cancer recurrence happens within 5 years^3, 4^. Recently, the inhibitors targeting poly (ADP-ribose) polymerase (PARP), such as rucaparib, have been developed and approved by the FDA for breast cancer associated protein (BRCA)-mutant ovarian cancer^5, 6^. Meanwhile, not all patients benefit from these drugs, and the expense of treatment is rather intolerable^7, 8^. Thus, developing new effective and affordable drugs is imperative.

Mangosteen (*Garcinia mangostana*) is a fruit that grows in tropical countries, such as Indonesia, Malaysia, and Thailand. Besides the arillus, the edible part, other parts of this plant, such as pericarps, barks, and roots, have been used as herbal medicines for hundreds of years in Southeast Asia^9^. In practical traditional Thai medicine, the pericarps of mangosteen were used to treat symptoms such as abdominal pain, leukorrhea, gonorrhea, and inflammation^10^. Current pharmacological research has revealed that the compounds isolated from the fruit hull exhibit antioxidant^11, 12^, anti-inflammatory^13, 14^, antinociceptive^15^, antitumor^16^, and anti-microbial^17^ effects. Among all secondary metabolites from mangosteen, xanthones were found to be one of the most effective antitumor components.

Xanthones are a class of polyphenolic compounds with a xanthene-9-one skeleton^18^. Recently, we isolated 24 xanthones from mangosteen pericarps and tested their anti-proliferative effects via 3-(4,5-dimethylthiazol-2-yl)-2,5-diphenyl tetrazolium bromide (MTT) assay. α-Mangostin is known as one of the primary xanthones, which has been thoroughly studied; however, we found that garcinone E (GE) exerted similar and possibly enhanced anticancer properties in the tested cancer cell lines. Although it demonstrated some anti-proliferative effects in several cancer cell lines^19–21^, its anticancer mechanism has not yet been well determined. Therefore, this study would be focused on the anti-ovarian cancer effects and mechanisms of GE.

## Results

### Anti-proliferative effects of xanthones in cancer cells

**Figure 1:**
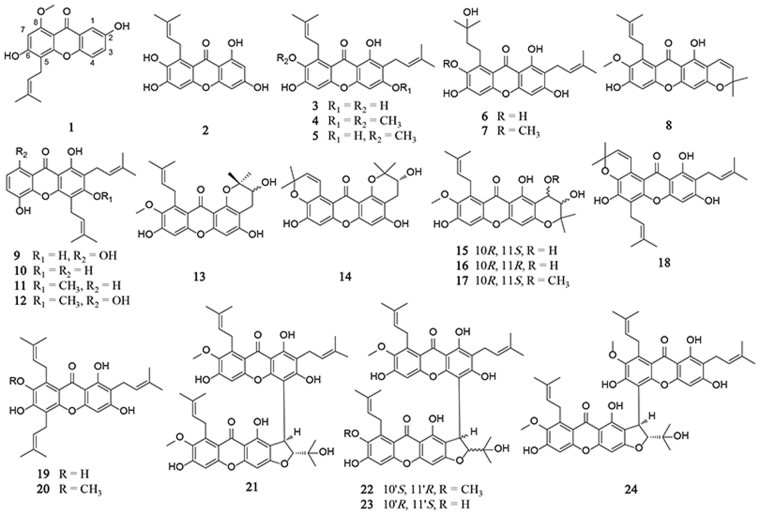
Chemical structures of the 24 xanthones.

Firstly, MTT assay was performed to evaluate the anti-proliferative activities of 24 xanthones isolated from mangosteen (Fig. 1A). The inhibition rates of cell viability in HEY and A549 cells are listed in Table 1 and Supplemental Table 1 after 24 h of the xanthone treatment. γ-Mangostin (**3**), β-mangostin (**4**), α-mangostin (**5**), garcinone C (**6**), 9-hydroxycalabaxanthone (**8**), 8-deoxygartanin (**10**), 8-hydroxycudraxanthone G (**12**), tovophyllin A (**18**), GE (**19**), and 7-*O*-methylgarcinone E (**20**) strongly inhibited cell viabilities in HEY cells, where the IC_50_s were less than 10 μM. The IC_50_ of GE in HEY cells was 7.79 ± 1.12 μM (24 h).

**Table 1:** IC_50_s in HEY cells of the isolated compounds.

No.	Compounds	IC_50_ (μM)/24 h
1	mangosharin	13.55 ± 4.97
2	1,3,6,7-tetrahydroxy-8-prenylxanthone	>20
3	γ-mangostin	9.31 ± 1.60
4	β-mangostin	7.74 ± 0.83
5	α-mangostin	9.14 ± 1.70
6	garcinone C	9.23 ± 3.08
7	garcinone D	11.05 ± 3.02
8	9-hydroxycalabaxanthone	7.35 ± 1.71
9	gartanin	11.46 ± 2.55
10	8-deoxygartanin	6.35 ± 0.15
11	cudraxanthone G	>20
12	8-hydroxycudraxanthone G	9.19 ± 2.09
13	11-hydroxy-1-isomangostin	>20
14	garcinoxanthone G	>20
15	garcinoxanthone E	>20
16	garcinoxanthone D	>20
17	garcinoxanthone F	>20
18	tovophyllin A	6.73 ± 2.78
19	garcinone E	7.79 ± 1.12
20	7-*O*-methylgarcinone E	6.52 ± 1.32
21	cratoxyxanthone	>20
22	garcinoxanthone B	>20
23	garcinoxanthone C	16.96 ± 4.44
24	garcinoxanthone A	>20

**Figure 2:**
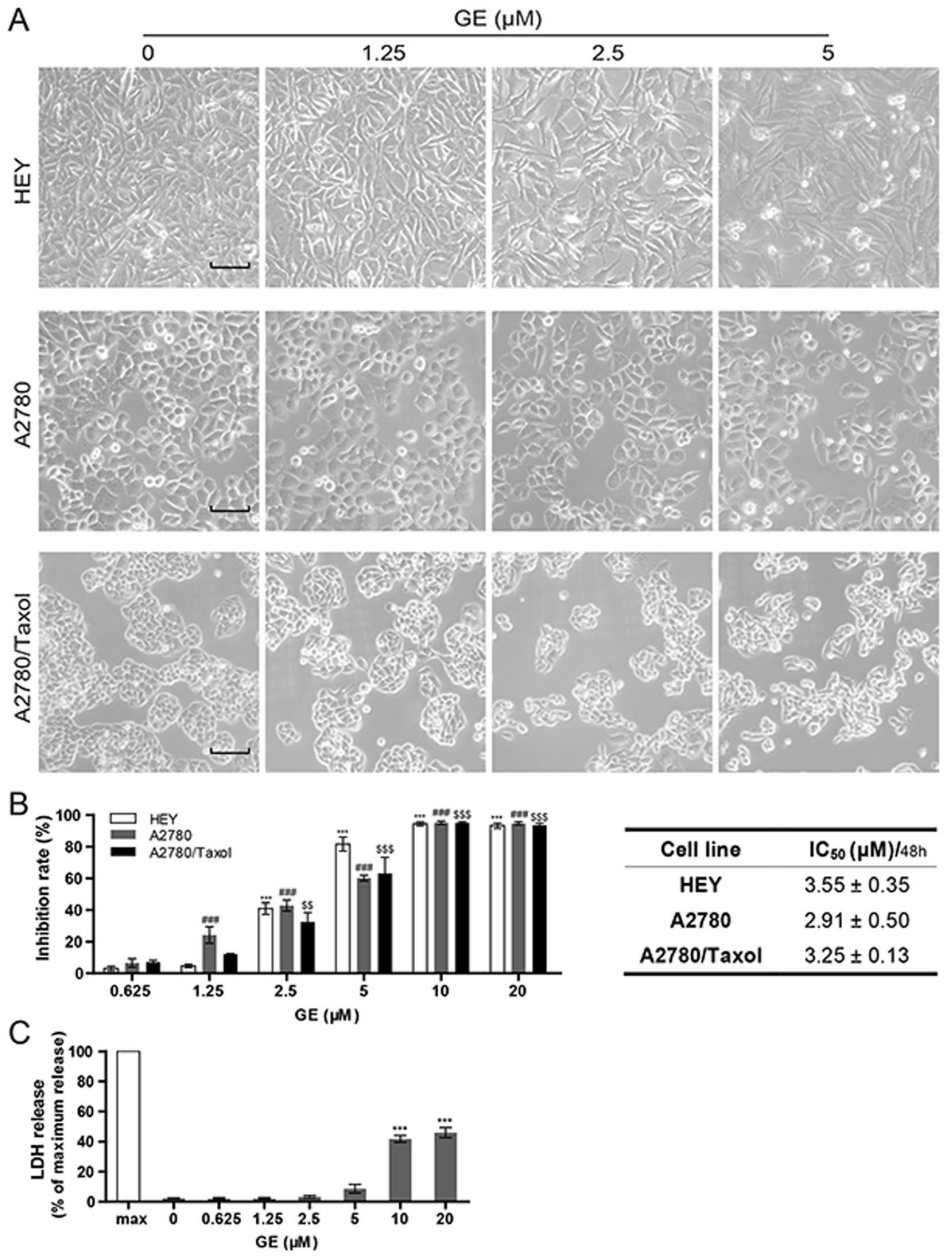
GE inhibited cell proliferation in ovarian cancer cells. (**A**) Morphological changes of HEY, A2780, and A2780/Taxol after 24 h of GE treatment. The length of the scale bar is 50 μm. (**B**) After 48 h of GE treatment, the inhibition rates of cell proliferation of three cell lines were tested by MTT assay. (**C**) The relative extracellular LDH concentration of HEY cells was tested by LDH release assay. All experiments were performed three times.

HEY is a malignant cell line originally derived from a patient with papillary serous cystadenocarcinoma with high potential of migration and invasion. Therefore, it was chosen as the model cells in most of experiments in this research. A2780, on the other hand, is a sensitive cell line cloned from an untreated patient and often used in anti-ovarian cancer study. It is the parental cell line of A2780/Taxol, A2780/CCP and other multidrug-resistant derivatives. To further evaluate the anti-ovarian cancer effects of GE *in vitro*, we performed several assays on three ovarian cancer cell lines: HEY, A2780, and A2780/Taxol (paclitaxel-resistant cell line). The cells were slender after treatment, and a few were suspended above the bottom of culture plates (Fig. 2A). After 48 h of GE treatment, the anti-proliferative effect was examined by MTT assay. Compared with the control group, GE significantly inhibited the cell viabilities of the cell lines with IC_50_s at 3.55 ± 0.35 μM (HEY), 2.91 ± 0.50 μM (A2780), and 3.25 ± 0.13 μM (A2780/Taxol). The resistance index on GE was 1.12 (Fig. 2B). These results suggest that not only did GE exhibit promising anti-proliferative effects, but it also demonstrated a similar activity in the multidrug-resistant cell line compared to its parental sensitive cell line, which indicated a potential anti-multidrug-resistant prospect in ovarian cancer cells.

As the concentration of lactate dehydrogenase (LDH) released into the extracellular environment corresponds to the extent of cell membrane damage, LDH release assay was used to determine the cytotoxicity of GE. The results shown in Fig. 2C demonstrating that over 10 μM of GE remarkably increased LDH leakage of HEY cells, suggested that high concentration of GE might be cytotoxic. Therefore, no more than 5 μM of GE was used in the following pharmacological evaluation.

### GE induced apoptosis via caspase signaling pathway in ovarian cancer cells

**Figure 3:**
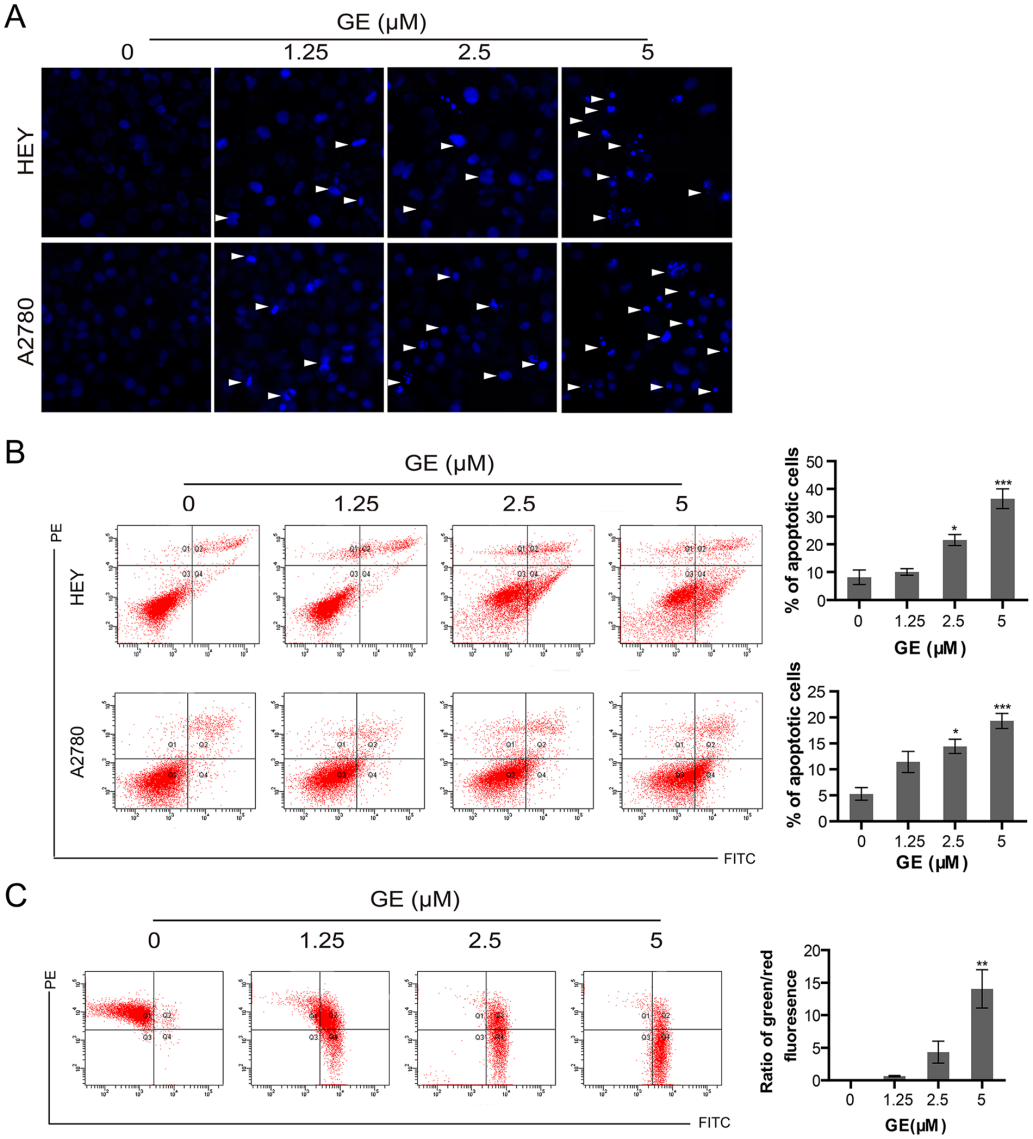
GE induced apoptosis in ovarian cancer cells. The nuclei of HEY and A2780 were stained with Hoechst 33342 (**A**), and the apoptotic cells of HEY and A2780 were examined by annexin V/PI staining assay (**B**). (**C**) The mitochondrial membrane potential of HEY cells was tested by JC-1 staining. All experiments were performed three times.

As shown in Fig. 3A, in HEY and A2780 cells, nuclei shrinkage and fracture appeared after 24 h GE treatment, suggesting that GE causes apoptosis in these cells. To count the number of apoptotic cells, annexin V/PI assay was used. After 24 h of GE treatment, the number of apoptotic cells in the treatment group was significantly higher than that in the control group. The percentages of apoptotic HEY cells in the 1.25, 2.5, and 5 μM- group were 10.07, 21.57, and 36.47, respectively; and in A2780 cells, the percentages were 11.43 (1.25 μM), 14.43 (2.5 μM), and 19.33 (5 μM). (Fig. 3B). Changes in mitochondrial membrane potential, which is considered a sign of early apoptosis, were evaluated by JC-1 staining. As shown in Fig. 3C, 3 h of GE treatment increased the green fluorescence and decreased the red fluorescence of HEY cells, indicating that the decline of mitochondrial membrane potential was caused by GE treatment.

**Figure 4:**
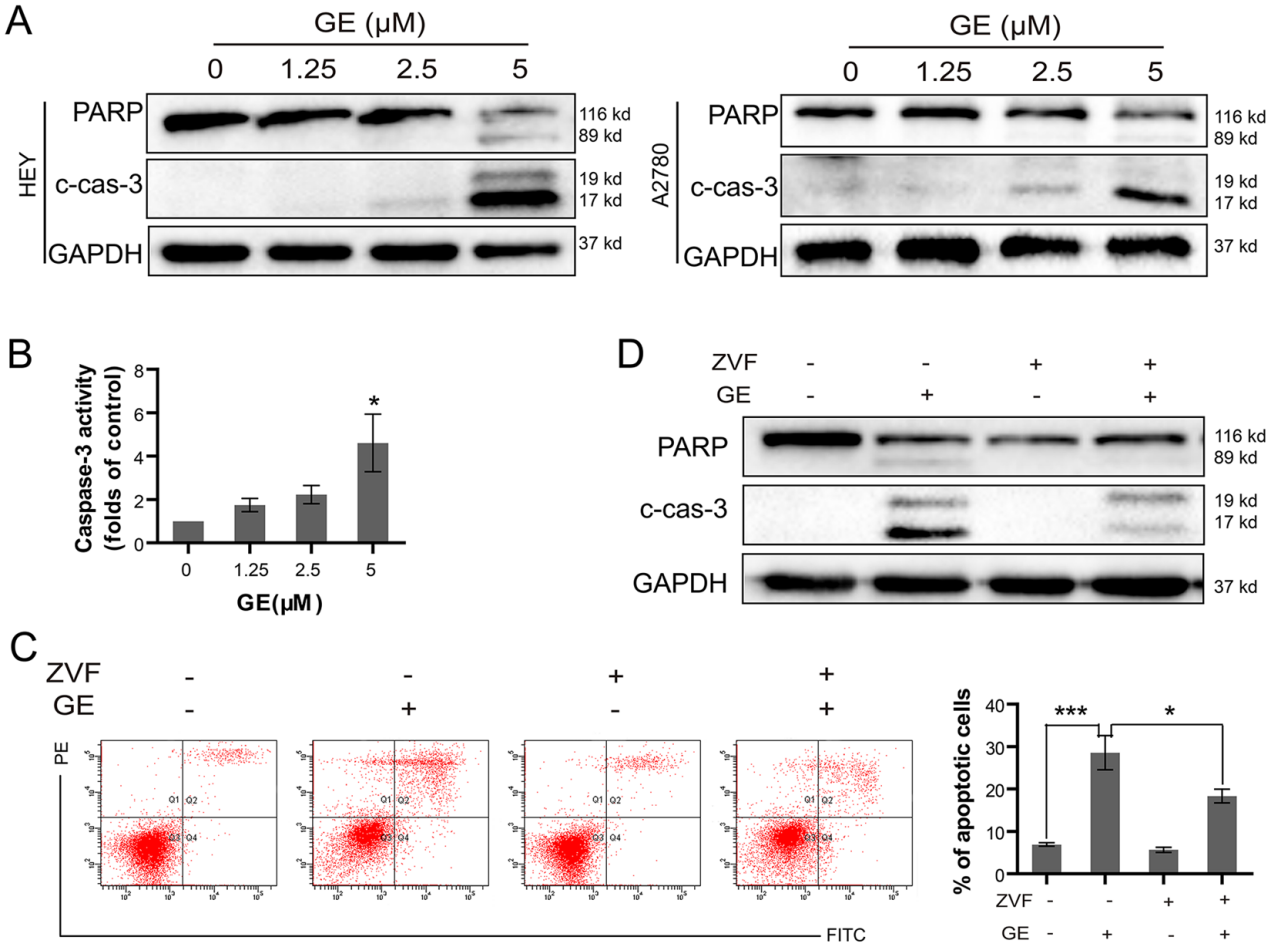
GE induced apoptosis via caspase signaling pathway. After 24 h of GE treatment, the apoptosis-associated protein in HEY and A2780 cells were examined by western blot (**A**), and caspase-3 activity in HEY cells was tested (**B**). After 1 h of ZVF and 24 h of GE treatment, the cells were examined by annexin V/PI staining assay (**C**), and the proteins were examined by western blot (**D**). The full-length gels of western blot were shown in the Supplementary Information file. All experiments were performed at least three times.

Subsequently, several apoptotic-related proteins were examined. After GE treatment, the protein levels of cleaved-caspase-3 were enhanced, which resulted in the enhancement of cleaved-PARP in HEY and A2780 cells (Fig. 4A). The activities of caspase-3 in HEY cells were also significantly upregulated (Fig. 4B). These results suggested that GE treatment induced apoptosis in ovarian cancer cells.

To further confirm that GE-induced apoptosis was caspase-dependent, a pan-caspase inhibitor, Z-VAD-FMK (ZVF), was used in pretreatment to block the cascade. As shown in Fig. 4C, ZVF pretreatment significantly decreased GE-induced apoptotic cells and attenuated the cleavage of caspase-3 and PARP (Fig. 4D), indicating that GE induces apoptosis via caspase signaling pathway in HEY cells.

### GE induced endoplasmic reticulum (ER) stress in ovarian cancer cells

**Figure 5:**
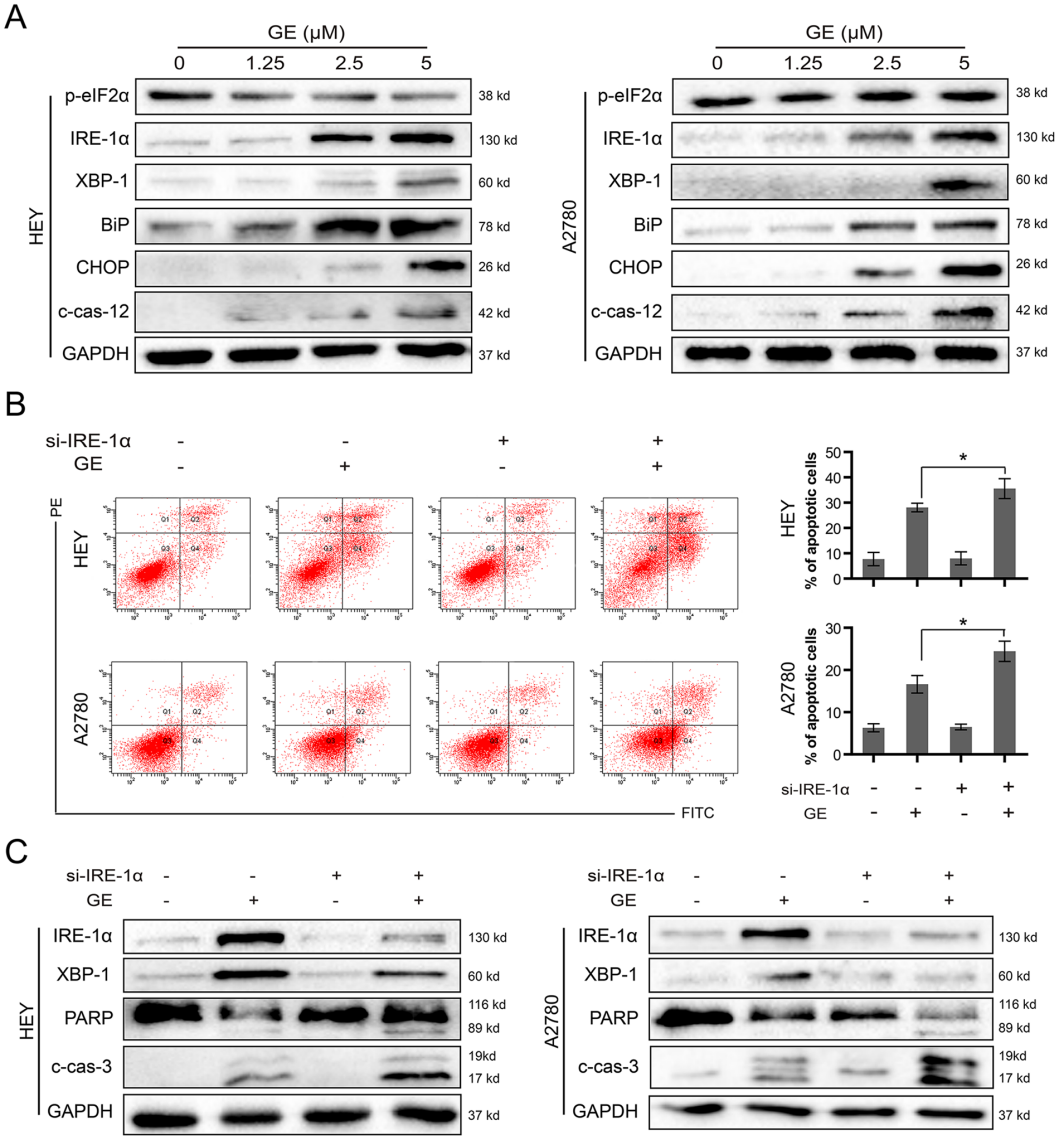
GE triggered ER stress and activated IRE-1α pathway in HEY and A2780 cells. (**A**) The related proteins were examined by western blot. After transiently transfected with scramble or IRE-1α siRNA and treated with GE for 24 h, the cells were examined by annexin V/PI staining assay (**B**), and the proteins were examined by western blot (**C**). The full-length gels of western blot were shown in the Supplementary Information file. All experiments were performed at least three times.

It has been reported that α-mangostin induces ER stress in cancer cells and promotes cell death^22^. As shown in Fig. 5A, GE showed no significant effects on phosphorylation eukaryotic translation initiation factor 2α (p-eIF2α), the downstream protein of protein kinase R-like ER kinase (PERK). Meanwhile, it dramatically increased the expression of inositol-requiring kinase (IRE)-1α, X-binding protein (XBP)-1, immunoglobulin-binding protein (BiP), and C/EBP homologous protein (CHOP) in HEY and A2780 cells, indicating it induces ER stress. Caspase-12 is located on the ER cytoplasmic surface and activated by ER stress pathways, which is considered as the key marker of ER stress-associated apoptosis^23^. In this study, the enhanced cleavage of caspase-12 was also observed in two cell lines after GE treatment. This result suggests that GE triggers ER stress-related apoptosis by inducing the cleavage of caspase-12, thus resulting in the activation of its downstream proteinase, caspase-3, followed by cell death.

### GE-induced IRE-1α activation exhibited cell protective effects

Interestingly, when the IRE-1α signaling pathway was triggered, IRE-1α activated XBP-1, the protein that increases the transcription of cell survival genes and protects the cells from lethal stimulation^24^. To determine the role of IRE-1α in the ovarian cells during GE treatment, IRE-1α in HEY and A2780 cells was knocked down by siRNA, and the number of apoptotic cells and levels of related proteins were checked. As shown in Fig. 5B and C, knocking down IRE-1α in HEY and A2780 cells caused more apoptotic cells and enhanced caspase-3 and PARP cleavage, indicating that activation of the IRE-1α signaling pathway partially protects the cells from death, whereas knocking down IRE-1α would further increase the apoptosis-induction effects of GE treatment.

### GE inhibited migration and invasion in ovarian cancer cells

**Figure 6:**
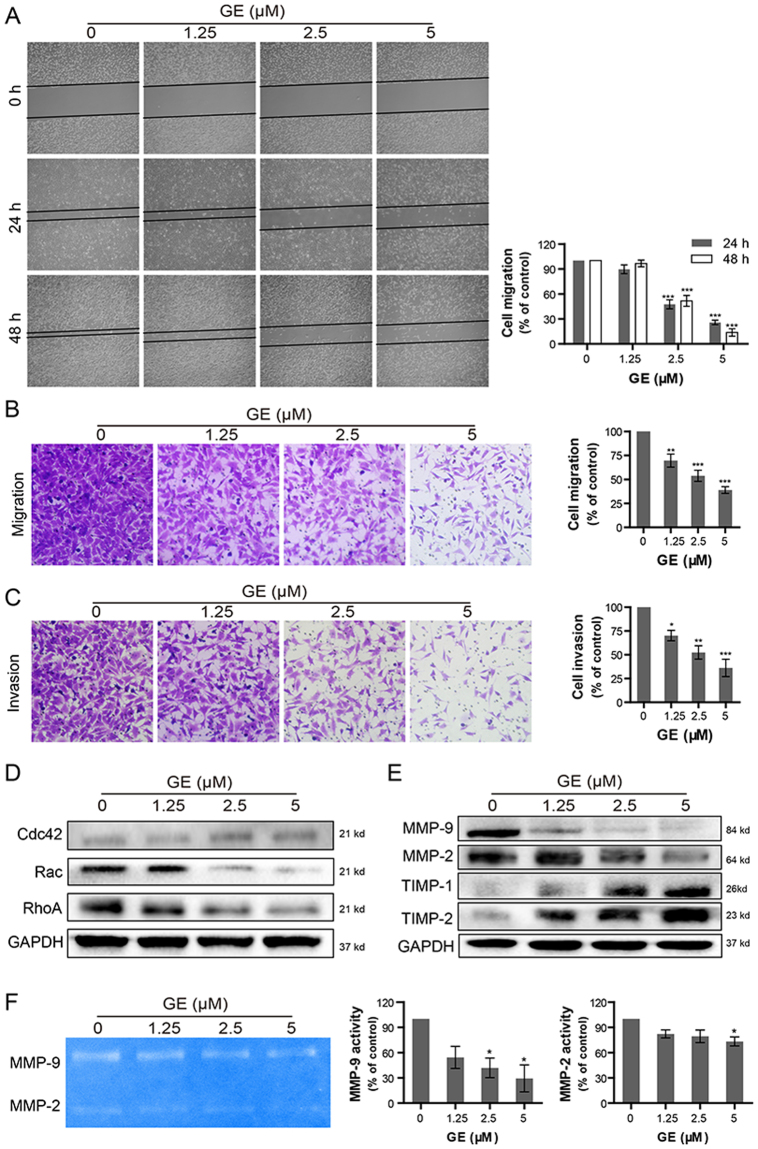
Inhibitory effects of GE on the cell migration and invasion of HEY cells. (**A**) The movement of HEY cells was observed by wound-healing assay at 0, 24, and 48 h after GE treatment. The migration and invasion abilities of HEY cells were examined by Transwell assay (**B, C**). Migration-associated proteins (**D**) and invasion-associated proteins (**E**) were evaluated by western blot. The activities of extracellular MMPs were determined by gelatin zymography assay (**F**). The full-length gels were shown in the Supplementary Information file. All experiments were performed at least three times.

One significant characteristic of ovarian cancer is high potential for metastasis, which involves cell migration and invasion during the process. HEY cells are highly malignant with strong migration and invasion abilities. To investigate whether GE exhibits anti-migration and anti-invasion effects, wound healing and Transwell assays were performed. As shown in Fig. 6A, the migratory ability of HEY cells was inhibited by GE treatment in a concentration- and time-dependent manner. The inhibitory effects detected by Transwell assay showed that treatments with 1.25, 2.5, and 5 μM GE downregulated 30%, 46%, and 61% of HEY cell migration, respectively, compared with the control group (Fig. 6B). From Fig. 6C, the number of invasive cells also significantly decreased in Matrigel-coated Transwell assay after GE treatment, and treatments with 1.25, 2.5, and 5 μM GE downregulated 30%, 48%, and 64% of HEY cell invasion, respectively.

Rho GTPases play an essential role in cell proliferation and migration. As shown in Fig. 6D, excluding Cdc42, the other two components of the Rho GTPase complex, RhoA and Rac, remarkably decreased after 24 h GE treatment, suggesting that GE might attenuate HEY cell migration by downregulating protein levels of RhoA and Rac.

Matrix metalloproteinases (MMPs) are integral in basement membrane degradation during the progress of tumor cell invasion, and tissue inhibitors of metalloproteinases (TIMPs) are the natural inhibitors of MMPs that suppress their activities. As shown in Fig. 6E, GE treatment was found to downregulate protein levels of MMP-9 and MMP-2 and upregulate protein levels of TIMP-1 and TIMP-2 at the same time, signifying that it inhibits HEY cell invasion not only by reducing the protein levels of MMPs but also by suppressing their activities. To confirm whether the vitalities of extracellular MMPs were attenuated after GE treatment, gelatin zymography assay was performed. As shown in Fig. 6F, the activities of MMP-9 and MMP-2 in the HEY culture medium remarkably decreased after GE treatment, demonstrating that GE inhibits HEY cell invasion by attenuating the activities of MMPs.

## Discussion

After careful analysis of the structural features of xanthones (**1**‒**24**) and their anti-proliferative potency, some regular structure-activity relationship patterns were summarized. (1) A hydroxyl group at C-1 and a 2-isoprenyl group at C-2 are necessary for the anti-proliferative activity of xanthones in cancer cells, such as compounds **3**‒**7**, **18**, and **19**. Among them, xanthones with an additional 2-isoprenyl group at C-8 (compounds **3**‒**5** and **19**) showed increased activities than others. (2) For xanthones containing two 2-isoprenyl groups at C-2 and C-4, the hydroxyl groups at C-1 and C-3 are essential for their anti-proliferative activity, such as compounds **9** and **10**. In summary, the hydroxyl group at C-1, and the 2-isoprenyl groups at C-2 and C-8 are essential for the anti-proliferative activity of xanthones in cancer cells.

The most abundant xanthone derivative in mangosteen pericarps is α-mangostin. *In vitro* and *in vivo* studies of α-mangostin show that it has many types of anticancer mechanisms including apoptosis-induction^16^, ER stress-stimulation^22^, autophagy-regulation^25^, and MMP-suppression^26^. In this study, GE exhibited comparable effective anticancer properties to α-mangostin under the same concentration that originally caught our attention. In ovarian cancer cells, GE presented remarkable anti-proliferative effects verified by MTT assay. The results of the LDH release assay showed that a high content (over 10 μM) of GE led to cell membrane failure and death. Thus, no more than 5 μM of GE were used in the following experiments. The results of Hoechst 33342 staining, annexin V/PI staining, JC-1 staining, and western blot revealed that GE induced apoptosis of ovarian cancer cells that caused the cleavage of caspase-3 and PARP, and it demonstrated stronger apoptotic-inducing effects than other xanthones in this study (data not shown). The blockage of caspase cascade by ZVF partially reversed cell apoptosis, indicating GE induced apoptosis partially by activating caspase pathway. In addition, GE might also cause cell death by other mechanisms, especially under a relatively high concentration. Moreover, GE retains similar activity in A2780/Taxol compared to the parental paclitaxel sensitive cell line A2780, and it downregulates the expression of P-gp in A2780/Taxol cells (Supplemental Fig. 1), suggesting it might show some prospects to overcome the multidrug-resistance of ovarian cancer cells, which needs to be further investigated. In addition, it caused more severe effects on autophagy than α-mangostin or other xanthones (data not shown). This evidence suggests that GE might be a promising candidate of anti-ovarian cancer drug. In the future, we might further compare the structure-activity relationship of GE and more xanthones derivatives, to screen for compounds with more anticancer efficiency and less toxicity to normal cells, and to develop a better drug delivery system to reduce toxicity and side effects to the body.

ER stress is induced when homeostasis of cells has failed. The function of ER is disordered under inter- or extracellular stimulation, which causes the accumulation of unfolded or wrongly folded proteins^27^. The subsequent unfolded protein response (UPR) will further activate IRE-1α, PERK, and activating transcription factor (ATF) 6 to promote degradation of accumulated proteins, which will determine the fate of the cells^28^. On one hand, the activation of IRE-1α will activate XBP-1, which promotes the expression of survival-associating genes and protects cells from stimulation^29, 30^. On the other hand, when the stimulation continues and UPR fails to restore intercellular homeostasis, the expression of downstream pro- apoptotic transcription factor, CHOP, will be triggered^31^, and pro-death crosstalk with the mitochondrial system will be mediated^24^. Subsequently, caspase-12 will be activated, thus activating caspase-3 and sentencing the cell to an apoptotic destiny^32^. In this study, we found that GE triggered ER stress that significantly enhancing the protein levels of IRE-1α, XBP-1, BiP, CHOP, and cleaved-caspase-12, and it showed a more intense ER stress-activation potential than most of xanthones included in this study. Previous study reported that both IRE-1α and PERK signaling pathways could be activated by α-mangostin^22^, whereas in our study, GE specifically triggered the IRE-1α pathway without enhancing the phosphorylation of eIF2α, the key downstream protein of PERK. Knocking down IRE-1α sequentially increased the number of apoptotic cells, and protein levels of cleaved-caspase-3 and cleaved-PARP, suggesting that the IRE-1α signaling pathway, to a certain extent, provided protection from GE-induced apoptosis. Similar protective effects of the IRE-1α signaling pathway have been reported^30, 33^. In this case, we assumed that though the GE-induced IRE-1α signaling pathway provided partial protection, its intensive continuous stimulation caused ER failure and ultimately, cell death. Meanwhile, blocking the pro-survival IRE-1α signaling pathway led the cells to more severe ER stress and ER collapse, which aggravated cell apoptosis.

The migratory ability is related to the metastasis potential of tumor cells, which contributes to cancer progression and poor outcomes for patients. The Rho family of GTPases plays essential roles in cell growth, division, and migration by modulating the actin cytoskeleton^34^. RhoA, Rac, and Cdc42 are the three most important members of Rho GTPases. Each member specifically regulates different mechanisms of cell mobility: RhoA regulates stress fiber formation and actomyosin contractility, Rac modulates the formation of lamellipodial protrusions and membrane ruffles, and Cdc42 triggers filopodial extensions at the cell periphery^35^. The results of wound-healing and Transwell (uncoated) assays suggested that GE not only inhibited idiopathic motion but attenuated chemotactic movement of HEY cells. Moreover, GE treatment was found to decrease the expression of RhoA and Rac. It is likely for GE to impair the migratory ability of HEY cells associated with suppressing stress fiber and lamellipodial protrusion formation that attenuating focal adhesion assembly, which inhibits the cells’ formation of anchored sites that used to move forward.

The extracellular matrix is the physical barrier preventing metastasis of tumor cells. The MMP family happens to be the most important proteinases that induce extracellular matrix degradation, thus, is closely related to tumor cell invasion^36, 37^. MMP-9 and MMP-2, which are highly expressed in metastatic tumors, are the only two type IV collagenases that are necessary prerequisites for cell invasion^38^. TIMPs exclusively inhibit the activities of MMPs. For example, TIMP-1 mainly inhibits MMP-9, and TIMP-2 suppresses MMP-2^39, 40^. In this study, we found that GE attenuated HEY cell invasion not only by downregulating the expression of MMP-9 and MMP-2 but also by upregulating protein levels of TIMP-1 and TIMP-2 to suppress MMPs activities. The result of gelatin zymography confirmed that the activities of MMP-9 and MMP-2 in the extracellular environment were remarkably restrained after GE treatment. Thus, the regulation of MMPs and TIMPs might be involved in the anti-invasive effect of GE. In addition, the proliferative reduction of the cells caused by GE treatment might also contribute to the inhibition effects of migration and invasion. It should be mention that A2780 cells don’t exhibit strong migration and invasion potentials, that even in the control group, most of the cells failed to move through the membranes in the Transwell assay. As a result, we didn’t further investigate the changes of the relative proteins in A2780 cells.

Taken together, GE effectively suppressed proliferation in ovarian cancer cells. It triggered ER stress that activated the IRE-1α signaling pathway, and knocking down IRE-1α would further induce apoptosis. Moreover, GE exhibited anti-migration effects associated to the inhibition of Rho GTPase expression, and attenuated cell invasion, which might be related to the modification of MMPs and TIMPs. Therefore, GE might be a prospective candidate as an anti-ovarian cancer agent, which requires further research.

## Materials and Methods

### General experimental procedures

Optical rotations were taken using a Perkin-Elmer 341 polarimeter (Waltham, MA, USA). IR spectra were recorded on a Bruker Tensor 37 infrared spectrophotometer using KBr disks (Billerica, MA, USA). The UV spectra were recorded on a JASCO V-650 spectrophotometer. The CD spectra were recorded on a JASCO *J*-815 spectropolarimeter (Easton, MD, USA). The NMR spectra were recorded on a Bruker 600 NMR spectrometer (Billerica, MA, USA). Chemical shift (*δ*) values were given in ppm with TMS as the internal standard, and coupling constants (*J*) were in Hz. ESIMS and HRESIMS spectra were recorded on an LTQ-Orbitrap XL spectrometer (Thermo Fisher Scientific, Waltham, MA, USA). All solvents were analytical grade (Tianjin Chemical Plant, Tianjin, China). Silica gel used for flash chromatography and precoated silica gel GF254 plates used for TLC were produced by Qingdao Haiyang Chemical Co., Ltd. The TLC spots were viewed at 254 nm and visualized by spraying with 5% H_2_SO_4_ in alcohol. Sephadex LH-20 gel (Amersham Biosciences, Piscataway, NJ, USA), ODS gel (12 nm, S-50 μm, YMC Co., Ltd.), and MCI gel (CHP20P, 75–150 μm, Mitsubishi Chemical Industries Ltd. Tokyo, Japan) were used for column chromatography (CC). Preparative HPLC was performed on a Shimadzu LC-20AP instrument with an SPD-M20A PDA detector (Tokyo, Japan). Chromatographic separations were carried out on a C_18_ column (250 × 19 mm, 5 μm, Waters, SunFire^TM^), using a gradient solvent system comprising H_2_O and CH_3_CN at a flow rate of 10 mL/min.

### Plant material

The pericarps of *G. mangostana* were collected in Chiang Mai, Thailand and identified by Professor Jingui Shen from Shanghai Institute of Materia Medica, Chinese Academy of Sciences. A voucher is deposited at the herbarium of the Institute of Chinese Medical Sciences, University of Macau (LL-20130901).

### Extraction and isolation

The extraction and isolation procedures were described previously^14^. Briefly, air-dried pericarps of *G. mangostana* were ground into powder and extracted with 30 L 95% alcohol at room temperature three times, for a total of three days. After evaporation of the collected percolate, the crude extract was suspended in 3.2 L H_2_O and partitioned with petroleum ether (2 L × 3 times), chloroform (3 L × 3 times), ethyl acetate (3 L × 3 times), and *n*-butanol (1 L × 3 times) successively. Around 200.0 g chloroform extract was subjected to thorough isolation by CC over silica gel, MCI, ODS, and Sephadex LH-20, as well as preparative HPLC, to obtain 24 xanthones, including mangosharin (**1**)^41^, 3,6,7-tetrahydroxy-8-prenylxanthone (**2**)^42^, γ-mangostin (**3**)^43^, β-mangostin (**4**)^44^, α-mangostin (**5**)^45^, garcinone C (**6**)^45^, garcinone D (**7**)^46^, 9-hydroxycalabaxanthone (**8**)^47^, gartanin (**9**)^48^, deoxygartanin (**10**)^49^, cudraxanthone G (**11**)^50^, 8-hydroxycudraxanthone G (**12**)^51^, 11-hydroxy-1-isomangostin (**13**)^52^, garcinoxanthones G (**14**), E (**15**), D (**16**) and F (**17**)^14^, tovophyllin A (**18**)^46^, garcinone E (**19**)^43^, 7-*O*-methylgarcinone E (**20**)^53^, cratoxyxanthone (**21**)^52^, and garcinoxanthones B (**22**), C (**23**) and A (**24**)^14^. The structures of the xanthones were identified by comparison of their observed and reported spectroscopic and physical data, and all structures were shown in Fig. 1A.

### Cell culture

Three human serous ovarian cancer cell lines, HEY, A2780, and A2780/Taxol were used as model cells. The HEY cell line was obtained from American Type Culture Collection (ATCC, Rockville, MD, USA), and A2780 cells and its taxol resistant cell line, A2780/Taxol cells were purchased from KeyGEN Biotech. HEY cells and A2780 cells were cultured in DMEM (GIBCO, Carlsbad, CA, USA), and A2780/Taxol cells were cultured in RPMI-1640 (GIBCO). All cell lines were supplemented with 10% FBS (GIBCO), 100 U/mL penicillin, and 100 μg/mL streptomycin (GIBCO) and maintained in a humidified incubator containing 5% CO_2_ at 37 °C. Exponentially growing cells were used for later experiments. The images of cell morphological characteristics were obtained using an Olympus IX73 microscope (Olympus, Tokyo, Japan).

### MTT assay

Each compound isolated from mangosteen pericarps was dissolved into 10 mM in DMSO to prepare the stock solution and then diluted with the culture medium to formulate the working solution. HEY, A2780, and A2780/Taxol cells were seeded into 96-well plates at a cell density of 4 × 10^3^ cells per well, cultured overnight, and treated with GE diluted in the medium containing 0.5% FBS for 24 or 48 h. The suspension was removed and replaced with 100 μL of 1 mg/mL MTT (Sigma, Saint Louis, MO, USA) solution. After 4 h of incubation, the suspension was removed, and 100 μL per well DMSO was added. Absorbance at 570 nm was determined by SpectraMax M5 microplate reader (Molecular Devices, Sunnyvale, CA, USA). The experiments in this assay were performed three times.

### LDH release assay

HEY cells were seeded into 96-well plates at a cell density of 4 × 10^3^ cells per well, cultured overnight, and treated with GE diluted in DMEM containing 0.5% FBS for 24 h. LDH release was examined by LDH cytotoxicity assay kit (Beyotime, Nantong, Jiangsu, China) in accordance with the manufacturer’s instructions. Absorbance at 450 nm was determined using a SpectraMax M5 microplate reader. Cell death rate was calculated as follows: cell death rate = 100 × (experimental release − spontaneous release)/(maximum release − spontaneous release). The value of maximum release (marked “max” in Fig. 2C) was tested with the treatment of maximum release solution in the kit. The experiments in this assay were performed three times.

### Hoechst 33342 staining

HEY and A2780 cells were seeded into 96-well plates at a cell density of 4 × 10^3^ cells per well, cultured overnight, and treated with GE diluted in the medium containing 0.5% FBS for 24 h. The suspension was removed, and the cells were fixed with 4% PFA (Sigma) for 20 min. 1 μg/mL of Hoechst 33342 (Sigma) was added and incubated for 15 min in the dark. Apoptotic morphological changes in the nucleus were observed using an In Cell Analyzer 2000 System (GE Healthcare, Uppsala, Uppsala, Sweden). The experiments in this assay were performed three times.

### Annexin V/PI assay

HEY and A2780 cells were seeded into 12-well plates at a cell density of 5 × 10^4^ cells per well, cultured overnight, and treated with GE diluted in DMEM containing 0.5% FBS for 24 h. The supernatants with the cells that were trypsinized by non-EDTA trypsin (GIBCO) were collected. Afterward, the cells were centrifuged, stained with Annexin V-FITC and propidium iodide (BioVision), and analyzed with a BD FACS Canto^TM^ flow cytometer (BD Biosciences, San Jose, CA, USA). The experiments in this assay were performed three times.

### JC-1 staining

HEY cells were seeded into 96-well plates at a cell density of 4 × 10^3^ cells per well, cultured overnight, and treated with GE diluted in DMEM containing 0.5% FBS for 3 h. After incubation was completed, the supernatants were removed, and the cells were stained with JC-1 (Beyotime) for 30 min at 37 °C. Next, the cells were suspended with trypsin, centrifuged, and analyzed with a BD FACS Canto^TM^ flow cytometer. The experiments in this assay were performed three times.

### Caspase-3 activity assay

HEY cells were seeded into 6-well plates at a cell density of 1 × 10^5^ cells per well, cultured overnight, and treated with GE diluted in DMEM containing 0.5% FBS for 24 h. Caspase-3 activity was examined by using a caspase-3 activity assay kit (Beyotime) in accordance with the manufacturer’s instructions. Absorbance at 405 nm was determined using SpectraMax M5 microplate reader. The experiments in this assay were performed three times.

### siRNA interference

The target sequence for IRE-1α and negative control (NC) were synthesized by GenePharma (Shanghai, China). HEY and A2780 cells were seeded into 6-well plates at a cell density of 8 × 10^4^ cells per well, cultured overnight, then transfected with IRE-1α and NC siRNA by lipofectamine^TM^ 2000 Transfection Reagent (Invitrogen Corp., Carlsbad, CA, USA) with serum-free DMEM. After 6 h incubation, the supernatants were removed and DMEM with 10% FBS was added followed by overnight incubation. The cells were treated with GE for 24 h and collected for annexin V/PI assay or western blot. The target sequences are as follows: (si-IRE-1α: sense: 5′-CUCCGAGCCAUGAGAAAUATT-3′, antisense: 5′-UAUUUCUCAUGGCUCGGAGTT-3′; NC: sense: 5′-UUCUCCGAACGUGUCACGUTT-3′, antisense: 5′-ACGUGACACGUUCGGAGAATT-3′). The experiments in this assay were performed at least three times.

### Wound-healing assay

HEY cells were seeded into 6-well plates at a cell density of 1 × 10^5^ cells per well and cultured overnight. Afterward, the monolayer of HEY cells was scraped by using a 200 μL micropipette tip and washed with PBS. GE diluted in DMEM was added to the wells and treated for 48 h. Photomicrographs were obtained using an Olympus IX73 microscope at 0, 24, and 48 h after the treatment was administered. The experiments in this assay were performed three times.

### Transwell assay

The migration and invasion of HEY cells were analyzed in Transwell chambers in accordance with previously described methods^54^. The cells were initially starved for 1 h with serum-free DMEM, and the upper chamber of the Transwell was coated with 100 μL of PBS (for migration assay) or 100 μL of 1:12 mixture of Matrigel (BD Biosciences) and PBS (for invasion assay), and the whole plates were incubated at 37 °C for 30 min. Subsequently, the remaining PBS was removed. Afterward, the cells were trypsinized, and 35,000 (for migration assay) or 50,000 (for invasion assay) cells per well were seeded into the upper chamber with DMEM containing 1% FBS and 0, 1.25, 2.5, and 5 μM of GE, and DMEM containing 10% FBS and 0, 1.25, 2.5, and 5 μM of GE was added to the lower chamber. After 24 h of treatment, the suspension and the cells on the top layer of the membrane were removed using cotton swabs and the cells that invaded the lower surface of the membrane were fixed with 4% PFA and stained with crystal violet (Beyotime). Photomicrographs were obtained using an Olympus IX73 microscope. The experiments in this assay were performed three times.

### Gelatin zymography assay

The protein content of the HEY cells’ supernatants was determined by using a BCA protein assay kit (Pierce, Rockford, IL, USA) after the samples were centrifuged at 4,000 × *g* at 4 °C. Equal amounts of protein from each sample were mixed with 5× loading buffer and loaded onto a 10% SDS-PAGE containing 0.1% gelatin. After electrophoresis was completed, the gels were rinsed thrice with 0.25% Triton X-100 for 2 h at room temperature and then incubated at 37 °C in the developing buffer (50 mM Tris-HCl pH 7.4, 5 mM CaCl_2_, 200 mM NaCl) for 48 h. The gels were stained with 0.5% Coomassie Brilliant Blue R-250 (Beyotime) for one h and subsequently de-stained in 50% methanol and 10% glacial acetic acid for 30 min. Images were obtained using ChemiDoc MP Imaging System (Biorad, Richmond, CA, USA), and quantification was performed with Image Lab 5.1 (Biorad). The experiments in this assay were performed three times.

### Western blot

HEY and A2780 cells were seeded into 6-well plates and treated with GE. After the treatment was administered, the cells were lysed in RIPA lysis buffer (Beyotime) in an ice bath for 20 min. Protein concentration was determined by utilizing a BCA protein assay kit. Total proteins (20 μg) were subjected to 8–12% SDS-PAGE, transferred onto polyvinylidene fluoride membranes, and blocked with 5% nonfat milk in PBS with Tween 20 (PBST) at room temperature for 1 h. The membranes were probed with specific primary antibodies against p-eIF2α, IRE-1α, BiP, CHOP, caspase-12, PARP, Bcl-xL, GAPDH, RhoA, Rac, Cdc42, MMP-9, MMP-2, TIMP-1, and TIMP-2 (Cell Signaling Technology, Beverly, MA, USA), and caspase-3 (Beyotime) overnight at 4 °C. The membranes were washed with PBST thrice and then incubated with anti-rabbit IgG or anti-mouse IgG with a HRP-linked antibody (Cell Signaling Technology). Equal protein loading was verified by probing with GAPDH antibody. Images were obtained using a ChemiDoc MP Imaging System, and quantification was performed with Image Lab 5.1. The experiments in this assay were performed at least three times.

### Statistical analysis

All data were expressed as mean values and standard deviation. Significance was analyzed using ANOVA and Tukey’s multiple comparison test by GraphPad Prism (Demo, Version 5). *P* value less than 0.05 (*p* < 0.05) was considered as statistically significant.

## Electronic supplementary material

Supplementary information
