# Supplementary material for: Garcinone E induces apoptosis and inhibits migration and invasion in ovarian cancer cells
Source: Sci Rep. 2017 Sep 6;7:10718. doi: 10.1038/s41598-017-11417-4 (PMC5587559; doi:10.1038/s41598-017-11417-4)

**Garcinone E induces apoptosis and inhibits migration and invasion in ovarian cancer cells**

Xiao-Huang Xu^1,#^, Qian-Yu Liu^1,2,#^, Ting Li^1^, Jian-Lin Liu^1^, Xin Chen^1^, Li Huang^1^, Wen-An Qiang^3,4^, Xiuping Chen^1^, Yitao Wang^1^, Li-Gen Lin^1,*^, Jin-Jian Lu^1,*^

^1^ State Key Laboratory of Quality Research in Chinese Medicine, Institute of Chinese Medical Sciences, University of Macau, Macao, China

^2^ Guangdong Medical Device Quality Surveillance and Test Institute, Guangzhou, Guangdong, China

^3^ Division of Reproductive Science in Medicine, Department of Obstetrics and Gynecology, Feinberg School of Medicine, Northwestern University, Chicago, Illinois, USA

^4^ Center for Developmental Therapeutics, Chemistry of Life Processes Institute, Northwestern University, Evanston, Illinois, USA

^#^Co-first author

*To whom correspondence should be addressed.

Dr. Jin-Jian Lu, State Key Laboratory of Quality Research in Chinese Medicine, Institute of Chinese Medical Sciences, University of Macau, Avenida da Universidade, Taipa, Macao. Email address: [jinjianlu@umac.mo](mailto:jinjianlu@umac.mo). Phone number: 853-88224674. Fax number: 853-28841358

Dr. Li-Gen Lin, State Key Laboratory of Quality Research in Chinese Medicine, Institute of Chinese Medical Sciences, University of Macau, Avenida da Universidade, Taipa, Macao. Email address: [ligenl@umac.mo](mailto:xpchen@umac.mo). Phone number: 853-88228041. Fax number: 853-28841358

**Supplementary Information**

**Supplemental Table 1.** IC_50_s in A549 cells of the isolated compounds.

| **No.** | **Compounds** | **IC_50_ (μM)/24 h** |
| --- | --- | --- |
| **1** | mangosharin | 8.28 ± 0.81 |
| **2** | 1,3,6,7-tetrahydroxy-8-prenylxanthone | > 20 |
| **3** | γ-mangostin | 7.90 ± 0.40 |
| **4** | β-mangostin | 6.94 ± 1.11 |
| **5** | α-mangostin | 7.59 ± 0.57 |
| **6** | garcinone C | 9.71 ± 1.26 |
| **7** | garcinone D | 11.76 ± 0.97 |
| **8** | 9-hydroxycalabaxanthone | 8.45 ± 0.69 |
| **9** | gartanin | 8.85 ± 0.37 |
| **10** | 8-deoxygartanin | 8.44 ± 0.64 |
| **11** | cudraxanthone G | > 20 |
| **12** | 8-hydroxycudraxanthone G | 14.07 ± 2.14 |
| **13** | 11-hydroxy-1-isomangostin | > 20 |
| **14** | garcinoxanthone G | > 20 |
| **15** | garcinoxanthone E | > 20 |
| **16** | garcinoxanthone D | > 20 |
| **17** | garcinoxanthone F | > 20 |
| **18** | tovophyllin A | 10.78 ± 0.92 |
| **19** | garcinone E | 6.20 ± 0.66 |
| **20** | 7-*O*-methylgarcinone E | 11.76 ± 3.32 |
| **21** | cratoxyxanthone | > 20 |
| **22** | garcinoxanthone B | > 20 |
| **23** | garcinoxanthone C | > 20 |
| **24** | garcinoxanthone A | > 20 |

After 24 hours of xanthones treatment, the inhibition rates of cell viabilities of human non-small cell lung cancer A549 cells were tested by MTT assay, and their IC_50_s were calculated. The experiments were performed three times.


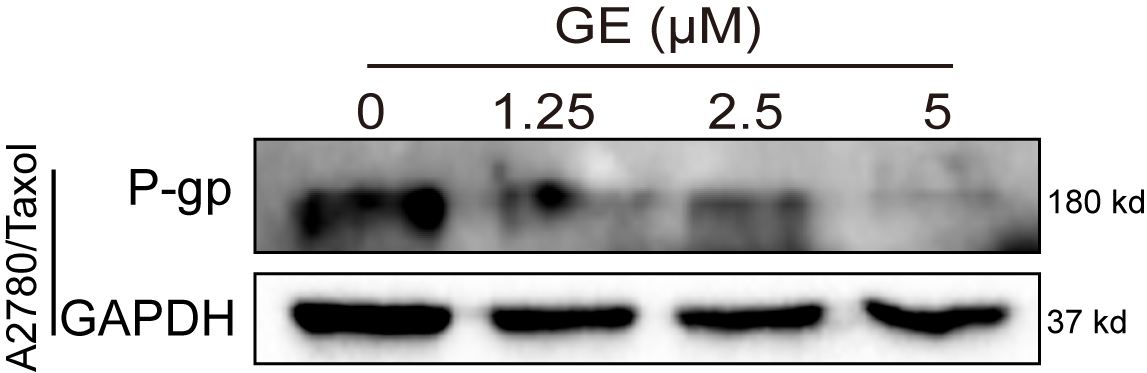


**Supplemental Figure 1. GE inhibited the expression of P-gp in A2780/Taxol.** After 24 hours of GE treatment, the protein level of P-gp in A2780/Taxol cells were tested by western blot. The experiments were performed three times.

The original data of western blot and gelatin zymography assay were presented as follows. And the results in the red frames were shown in the manuscript


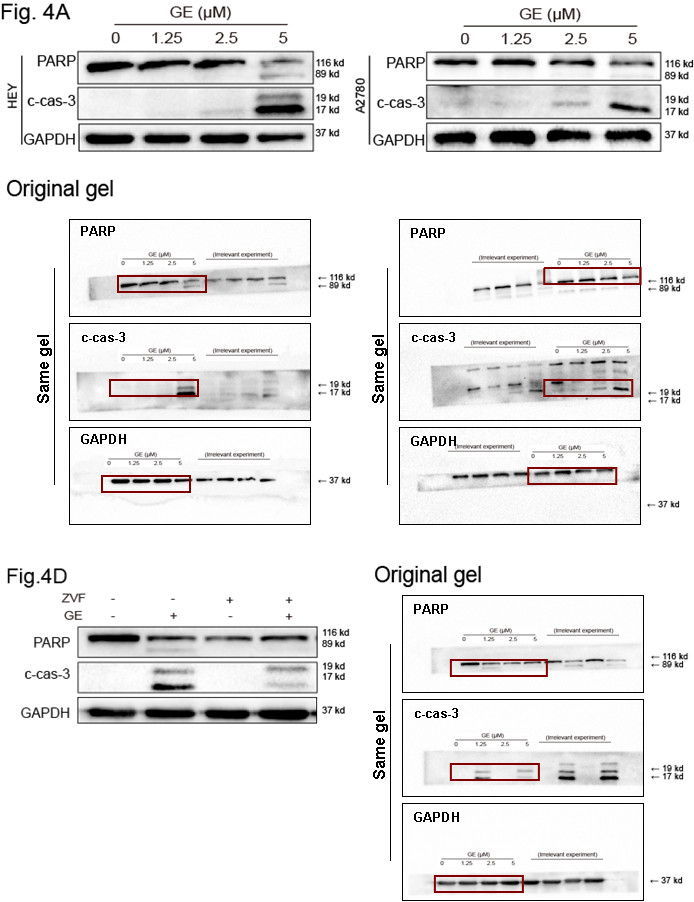


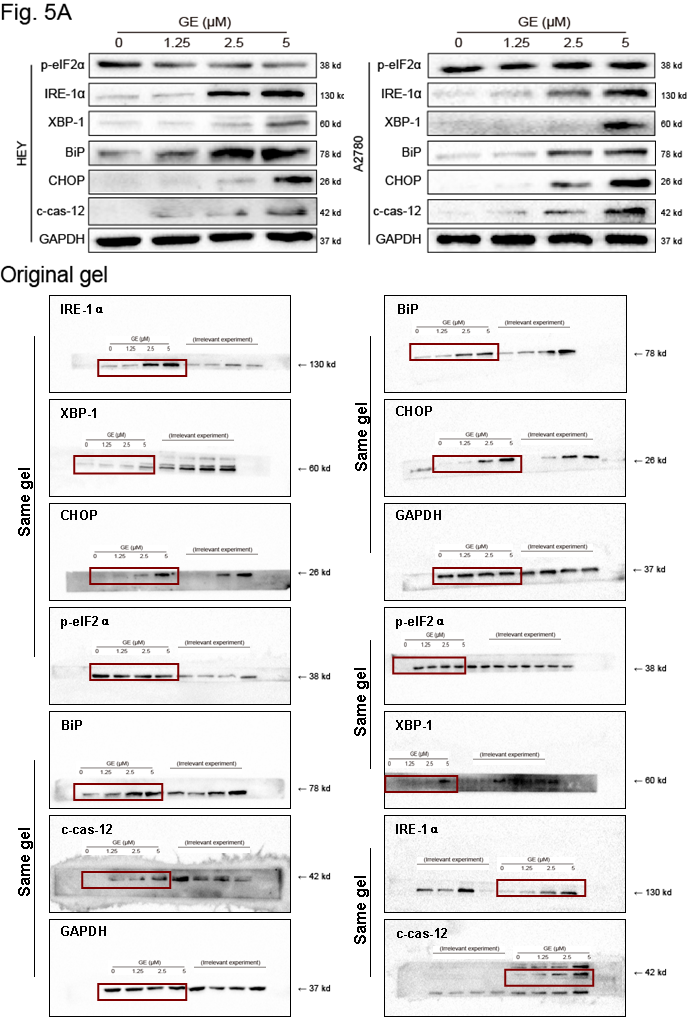


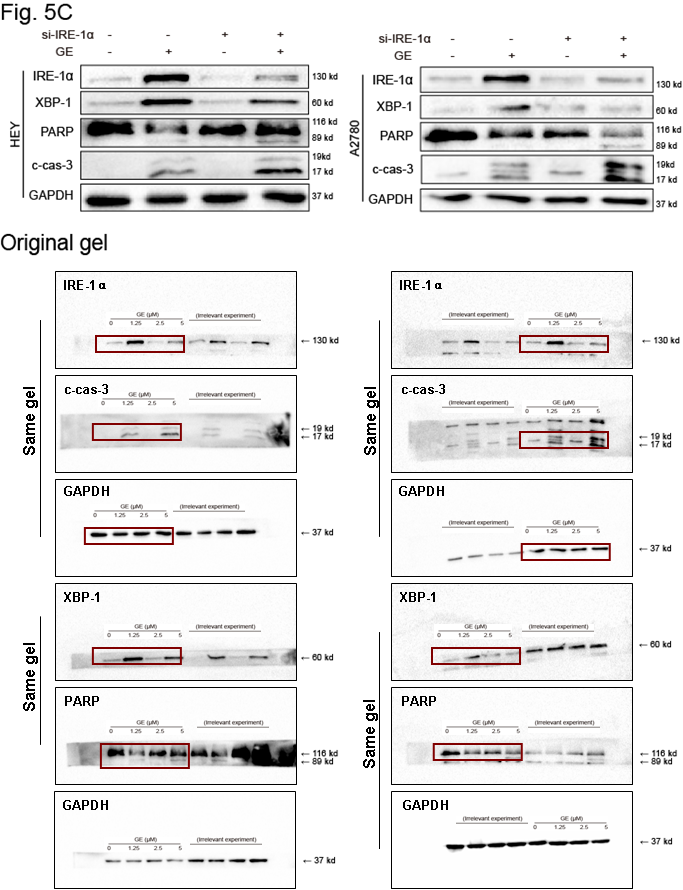


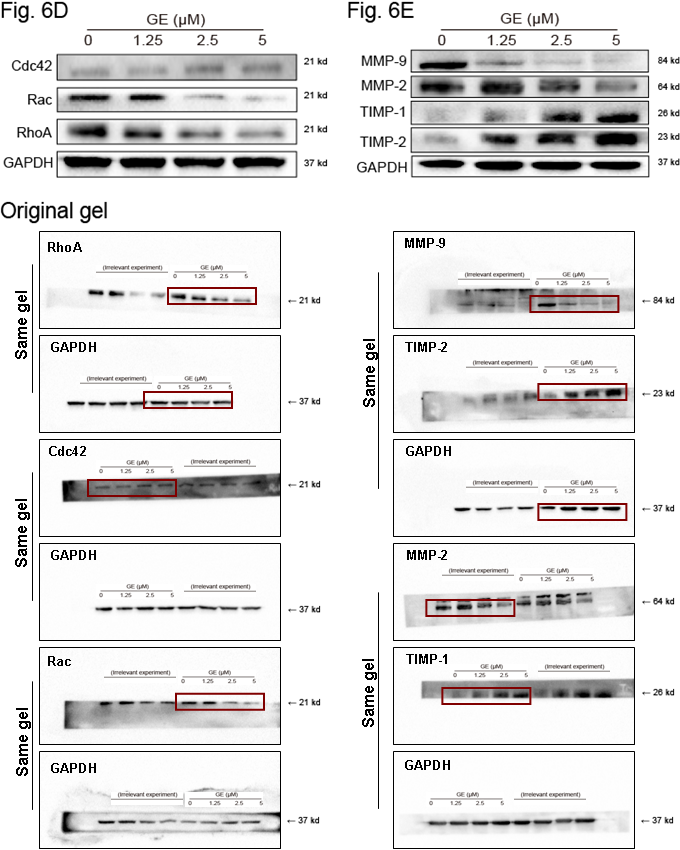


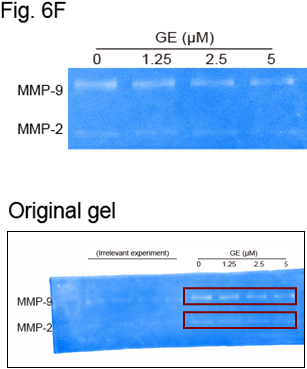

Supplement: Supplementary file 1 — Supplementary information [file 41598_2017_11417_MOESM1_ESM.doc]
